# Mechanisms and potential therapeutic targets of SphK1 and SphK2 in hepatocellular carcinoma

**DOI:** 10.3389/fmed.2025.1617401

**Published:** 2025-10-29

**Authors:** XiaoYing Xu, HengFei Li, RuiSi Li, Yue Xu, YiHeng Xu, HaiTang Huang, XiaoJuan Lv, Chu Liao, JunQiu Ye, Liu Bo

**Affiliations:** ^1^Hubei University of Chinese Medicine, Wuhan, China; ^2^National Traditional Chinese Medicine Disease Prevention and Control Base (Hubei), Wuhan, Hubei Province, China; ^3^Department of Hepatology, Hubei Key Laboratory of the Theory and Application Research of Liver and Kidney in Traditional Chinese Medicine, Hubei Provincial Hospital of Traditional Chinese Medicine, Wuhan, China; ^4^Affiliated Hospital of Hubei University of Chinese Medicine, Wuhan, China; ^5^Hubei Province Academy of Traditional Chinese Medicine, Wuhan, China

**Keywords:** HCC, S1P, SPHK1, SPHK2, mechanism

## Abstract

Hepatocellular carcinoma (HCC) is the third leading cause of cancer-related deaths globally. Sphingosine-1-phosphate (S1P) is catalyzed by sphingosine kinases SphK1 and SphK2 and plays a key role in HCC progression: SphK1 can drive tumor proliferation, migration, and angiogenesis. It activates the PI3K/AKT/mTOR and MAPK/ERK signaling pathways and mediates chemoresistance and immune suppression; SphK2 enhances histone acetylation and upregulates pro-oncogene expression through nuclear S1P. It also maintains telomere activity via mitochondrial S1P, which promotes tumor survival and facilitates resistance to regorafenib. In targeted therapy, SphK1 inhibitors (e.g., PF-543) and SphK2 inhibitors (e.g., ABC294640) have shown significant anti-tumor effects in preclinical models. Future research should focus on elucidating the regulatory networks of SphK1/SphK2 in different HCC subtypes, developing highly selective inhibitors, and advancing clinical trials based on metabolic-immune interaction regulation. This paper systematically summarizes the mechanisms of action and therapeutic progress of SphK1/SphK2 in HCC. It provides an important theoretical basis for the clinical translation of precision therapy strategies in HCC.

## 1 Introduction

Hepatocellular carcinoma (HCC) is the most common type of primary liver cancer. It accounts for 90% of all primary liver cancer cases (1). HCC has a high mortality rate; according to the 2020 global cancer statistics by the World Health Organization (WHO), liver cancer ranks sixth in terms of incidence and third in terms of mortality among all cancers. HCC has become the third leading cause of cancer-related deaths globally, after lung and colorectal cancers (2). The incidence and mortality rates of HCC exhibit similar geographical patterns, with higher rates observed in Asia and Africa (3). Due to its complex pathophysiological mechanisms, high metastasis and recurrence rates, HCC is considered a major public health issue. The main risk factors for HCC include hepatitis B virus (HBV) and hepatitis C virus (HCV) infections, obesity, and metabolic syndrome (4).

Liver function relies on the ability of mitochondria to generate ATP and their detoxification properties. Evidence suggests that liver diseases are characterized by severe mitochondrial dysfunction. High levels of mitochondrial DNA (mtDNA) have been found in the plasma of patients and mice with non-alcoholic steatohepatitis (NASH), and these mtDNA fragments have the ability to activate Toll-like receptor 9 (TLR9), leading to the inflammation phenotype characteristic liver disease (5). Under mitochondrial stress conditions, the mitochondrial permeability transition pore (mPTP) opens, leading to mitochondrial dysfunction (6). Extensive mtDNA damage exacerbates mitochondrial oxidative stress, which further damages hepatocytes. mtDNA also plays a crucial role in HBV-related liver injury and the development of hepatocellular carcinoma (HCC) (7).

Sphingosine kinase (SPHK) is an ATP-dependent lipid kinase that catalyzes the conversion of sphingosine (Sp) to sphingosine-1-phosphate (S1P). Two distinct SPHK isoforms have been identified to date: sphingosine kinase 1 (SPHK1) and sphingosine kinase 2 (SPHK2). Studies have shown that these isoforms play a potential oncogenic role in the development and progression of cancer (8). In the presence of ATP, S1P is synthesized by the phosphorylation of sphingosine (9). As a versatile lipophilic mediator, S1P is involved in various biological functions, including autoimmunity, inflammation, cardiovascular regulation, the central nervous system, diabetes, cell cycle regulation, and cancer (10). There is a known correlation between cancer and changes in sphingolipids, particularly the production of S1P by SPHK1 and SPHK2. However, the potential relationship between the bioactive lipid molecule SPHK and the cellular response to DNA damage remains unclear. Studies suggest that S1P may regulate the cellular response to DNA damage (DDR) (11). Dysregulation of DDR is considered one of the key mechanisms underlying the development of HCC. As an important signaling molecule, changes in S1P signaling pathways may affect DDR pathways (12). S1P indirectly modulates DNA stability by regulating cell-signaling and DNA-damage-repair pathways, without directly binding DNA.

In summary, by controlling S1P production, SphK1 and SphK2 tune oncogenic signaling networks; their sustained over-expression fuels the initiation and progression of multiple human cancers. Therefore, SphK1, SphK2, and their product S1P have become potential therapeutic targets for the treatment of various diseases, especially cancer cells.

## 2 Basic functions of SphK1 and SphK2

Sphingolipids, as essential lipids, play a broad role in cell structure and signaling. Sphingosine-1-phosphate (S1P) is a simple yet powerful bioactive sphingolipid molecule. As a key signaling molecule, it is widely involved in regulating various cellular processes. Additionally, S1P plays a significant role in the development of several pathological conditions, such as inflammatory diseases and cancer, by mediating complex signaling pathways (13).

### 2.1 Synthesis and metabolism of S1P

S1P is produced intracellularly by two sphingosine kinases, SphK1 and SphK2. A variety of bioactive molecules can rapidly activate SPHK1, including growth factors, hormones, pro-inflammatory cytokines, lipopolysaccharides (LPS), IgE/IgG receptor ligands, and various G protein-coupled receptor (GPCR) agonists, leading to the phosphorylation of sphingosine to generate S1P (14). Once activated, it undergoes significant subcellular localization changes, translocating from the cytoplasm to the plasma membrane (15). This translocation process mainly relies on the specific phosphorylation of the Ser225 residue in SphK1 by extracellular signal-regulated kinase (ERK) (16). The membrane translocation of SphK1 has important biological significance: it enables the kinase to be in proximity to sphingosine at the plasma membrane, and it allows for localized production of S1P near cell surface receptors, thus enabling “inside-out” signaling of S1P (17).

In contrast to SphK1, the activation mechanism of SphK2 has not been fully elucidated. Research shows that SphK2 has unique subcellular localization characteristics, primarily distributed in the cell nucleus and mitochondrial inner membrane. This compartmentalized distribution gives SphK2 a diverse range of biological functions. In the nucleus, SphK2 participates in gene transcription regulation by catalyzing the production of S1P. In mitochondria, SphK2 is the primary isoform responsible for S1P production. To clarify the precise localization of SphK2 in mitochondria, researchers have used digitonin to selectively disrupt the mitochondrial outer membrane. Experimental results indicate that SphK2 does not attach to the outer mitochondrial membrane. It is primarily located in the inner mitochondrial membrane (18). One study found that the subcellular localization of SPHK2 is controlled by a precise post-translational modification regulatory network, where protein kinase D (PKD)-mediated phosphorylation plays a key role. Specifically, when HeLa cells are stimulated by the tumor promoter phorbol 12-myristate 13-acetate (PMA), a novel putative nuclear export signal (NES) motif within SPHK2 is specifically phosphorylated. This key post-translational modification event triggers the active translocation of SPHK2 from the nucleus to the cytoplasm, thereby dynamically regulating its subcellular localization (19).

### 2.2 Biological functions of S1P

S1P forms a complex signaling network through its G protein-coupled receptors (S1PR1-S1PR5), playing pleiotropic regulatory roles in physiological and pathological processes. In HCC, S1P activates the Hippo pathway downstream effector YAP via S1PR2, inducing the expression of cysteine-rich protein 61 (CYR61) and connective tissue growth factor (CTGF), thereby driving abnormal tumor cell proliferation (20). This mechanism reveals the critical role of the S1P/S1PR2 axis in HCC progression. Furthermore, the dual signaling mode of S1P extends its functions: intracellularly, S1P regulates calcium homeostasis and forms a “sphingolipid rheostat” with ceramide, dynamically balancing cell survival and apoptosis; extracellularly, as a ligand for S1PR1–5, it activates PI3K/Akt, Rho/Rac, and ERK pathways, coordinating VEGFR/PDGFR to regulate angiogenesis and immune cell migration (21, 22). S1P promotes the secretion of vascular endothelial growth factor (VEGF) through S1PR1/S1PR3, playing a central role in tumor angiogenesis (23) while circulating S1P relies on S1PR1 to maintain endothelial barrier integrity and inhibit inflammatory vascular leakage (24). Recent studies also discovered that S1P, containing only double bonds, acts as an essential cofactor for TRAF2, activating the NF-κB pathway through K63 ubiquitination modification of RIP1, specifically inhibiting apoptosis. This provides a new perspective on enhancing anti-tumor efficacy by targeting the S1P-TRAF2 axis (15). In summary, the S1P/S1PR signaling network regulates cell fate, the immune microenvironment, and angiogenesis through multiple dimensions, making it a potential target for the treatment of liver cancer and other diseases.

**Table 1:** 

S1PR subtype name	Disease	Mechanism	quote
S1PR1	Graft-versus-host disease (GVHD)	The Sphk1/S1P/S1PR1 pathway specifically enhances mitochondrial fission and quality in allogeneic CD4 + T cells by activating AMPK/AKT/mTOR/Drp1	PMID:36071219
	Atherosclerosis	The anti-inflammatory effect of 17,18-EEQ involves activation of the S1PR1-Gq-Ca2 + -eNOS axis in endothelial cells; eNOS activation is abolished when S1PR1 or Gq signaling is inhibited.	PMID: 38907081
	Lung adenocarcinoma (LUAD)	S1PR1 regulates malignant functions of LUAD cells by inhibiting expression of COL5A1, MMP1, and SERPINE1 via the p-STAT1/miR-30c-5p/FOXA1 signaling pathway	PMID: 39551792
	Ovarian cancer	The S1PR1-PDK1-LATS1/2-YAP pathway regulates ovarian cancer cell senescence and is controlled through a YAP-mediated feedback loop	PMID: 37828220
	Triple-negative breast cancer (TNBC), Chemotherapy-induced peripheral neuropathy (CIPN)	Targeting the S1P/S1PR1 axis with FTY720	PMID: 39126272
	Hepatocellular carcinoma (HCC)	SMYD3 promotes HCC by upregulating S1PR1 expression via H3K4me3	PMID:34400616
		miR-363 directly targets the 3’-UTR of S1PR1 mRNA, inhibiting ERK and STAT3 activation	PMID: 24631531
	hepatic fibrosis	Human hMFs participate in liver fibrosis through the S1P/S1PRs signaling pathway	PMID: 21145832
		The SphK1/S1P/S1PR1/3 axis induces Ang1 expression through the S1PR1 and S1PR3 mediated signaling pathways.	PMID: 23466305
	Human acute myeloid leukemia (AML)	S1PR1 regulates multiple MAPK signaling cascades, promoting cell survival and proliferation by enhancing MKK1-ERK signaling activation	PMID: 27572094
	Esophageal squamous cell carcinoma (ESCC)	S1PR1 promotes proliferation and reduces apoptosis by enhancing and directly activating p-STAT3	PMID: 31438989
	Colorectal cancer (CRC)	BATF3 strengthens proliferation, invasion, and metastasis of CRC cells by activating the S1PR1/p-STAT3/miR-155-3p/WDR82 axis	PMID: 33057139
	Pancreatic cancer	FTY720 inhibits Sonic-hedgehog (Shh) signaling mediated by the S1PR1/STAT3 loop in pancreatic cancer	PMID: 30083262
S1PR2	Inflammatory bowel disease (IBD)	The S1PR2/RhoA/ROCK1 signaling pathway participates in IBD pathogenesis by regulating vascular endothelial barrier function and M1 macrophage polarization	PMID: 35537530
	Liver ischemia–reperfusion injury (IRI)	IR stress coordinates hepatic bile acid metabolism producing TβMCA, which alleviates liver inflammatory damage by inhibiting the myeloid S1PR2-GSDMD axis	PMID: 39091991
	Sepsis	S1PR2 activates ROCK I to induce Drp1 phosphorylation, leading to Drp1-dependent mitochondrial fragmentation in macrophages.	PMID: 39626018
	Severe acute pancreatitis (SAP)	S1PR2 activation triggers pyroptosis in THP-1 cells via the RhoA/ROCK signaling pathway	PMID: 38827741
	Colorectal cancer (CRC)	Activation of S1PR2 upregulates DPD expression via the Hippo/TWIST1-JMJD3 pathway	PMID: 39814224
		JTE-013 exhibits anti-5-FU resistance activity by blocking S1PR2 internalization to the ER, thereby inhibiting 5-FU degradation to FBAL through downregulation of tumor DPD expression	PMID: 32088343
	hepatic fibrosis	S1P stimulated the activation of HSCs and liver fibrosis via S1PR2-mediated signaling.	PMID: 39236934
	Alzheimer’s disease (AD)	S1PR2 inhibits autophagy by activating the AKT/mTOR pathway	PMID: 38007654
	Cholangiopathies	ERK1/2 acts as a downstream regulator of S1PR2 signaling in human cholangiocytes and directly promotes reactive phenotype activation	PMID: 38407207
S1PR3	TAL1 T-cell acute lymphoblastic leukemia (T-ALL)	In T-ALL cells, TAL1-RUNX1 upregulates S1PR3 expression by binding to enhancer regions of the S1PR3 gene	PMID: 37591940
	Osteosarcoma (OS)	The S1P/S1PR3 axis inhibits YAP phosphorylation and promotes YAP nuclear translocation	PMID: 30587459
	Lymphangioleiomyomatosis (LAM)	Inhibition of SPHK1/S1PR3 signaling triggers autophagic cell death in TSC2-deficient cells	PMID: 36543771
	Renal cell carcinoma (RCC)	S1P induces cell proliferation via the S1PR3/Gi/p38/Akt/p65/cyclin D1-CDK4 pathway and migration through S1PR3/Gi/q/ERK/p38/p65	PMID: 35346818
	Psoriasis	S1PR3 functions via Gαi/PKA-mediated Src activation, enhancing STAT3 phosphorylation and subsequent transcriptional activity	PMID: 39833165
	Radiation-induced pulmonary fibrosis (RIPF)	Overexpression of miR-495-3p inhibits the S1PR3/SMAD2/3 pathway and suppresses the EMT process.	PMID: 31489649
	Lung adenocarcinoma (LUAD)	S1PR3 levels are upregulated via the TGF-*β*/SMAD3 signaling axis	PMID: 27856637
	Traumatic spinal cord injury (SCI)	The S1P/S1PR3/p38 MAPK pathway contributes to NF-κB p65 inflammatory response.	PMID: 33602274
	Undifferentiated nasopharyngeal carcinoma (NPC)	S1PR3 knockout inhibits AKT activation and S1P-induced NPC cell migration	PMID: 28240350
S1PR4	Nonalcoholic steatohepatitis (NASH)	S1P increased S1pr4 expression in hepatic macrophages and activated the NLRP3 inflammasome through inositol trisphosphate/inositol trisphosphate-receptor-dependent calcium signaling.	PMID: 34890841
	Head and neck squamous cell carcinoma (HNSCC)	Upregulation of S1PR4 enhances T cell function during CAR-T cell therapy	PMID: 37680482
	Psoriasis	S1pr4 signaling synergizes with TLR signaling in resident macrophages to produce CCL2	PMID: 32017036
	Rheumatoid arthritis (RA)	S1PR4 silencing may inhibit proliferation, migration, and proinflammatory responses and promote apoptosis of RA-FLSs partly by repressing IL-17	PMID: 36068391
	Osteosarcoma	S1PR4 activates the STAT3 signaling pathway to promote angiogenesis in HUVECs	PMID: 38767243
	Breast cancer	S1PR4 affects CD8 + T cell proliferation and survival intrinsically via expression of Pik3ap1 and Lta4h	PMID:32663191
	Prostate cancer	Toll-like receptor 4 (TLR4, LPS receptor)-sphingosine kinase 1 (SphK1) signaling	PMID: 31152147
	Skin cutaneous melanoma (SKCM)	S1PR4 improves prognosis of melanoma patients by enhancing CD8 + T cell transformation and activation	PMID: 38468937
S1PR5	Colon adenocarcinoma (COAD)	Downregulation of S1PR5 improves CRC response to anti-PD1 therapy	PMID: 38312843
	Cerebral ischemia/reperfusion (CI/R) injury	A-971432 attenuates CI/R injury-induced apoptosis and inflammation through PI3K/Akt and MAPK pathways	PMID: 40262248
	Chronic obstructive pulmonary disease (COPD)	There is a correlation between expression levels of receptors and enzymes involved in pulmonary sphingosine kinase signaling systems	PMID: 21945191
	Acute kidney injury	The JAK2/STAT3 pathway participates in the I/R kidney model	PMID: 39155330

## 3 The role of SphK1 and SphK2 in HCC mechanisms

Sphingolipid metabolites have gained significant attention as key regulatory molecules in cancer diagnosis and treatment. Progression from chronic liver disease to hepatocellular carcinoma (HCC), there are significant dynamic changes in the serum sphingolipid profile. Compared to patients with cirrhosis, the serum levels of C16-ceramide (C16-Cer) and S1P are significantly elevated in HCC patients, suggesting that these molecules could serve as potential diagnostic markers for the malignant transformation of liver disease (25). The core molecules of sphingolipid metabolism—ceramide (Cer) and S1P—regulate cell fate through the “sphingolipid rheostat” model: Cer inhibits tumor growth by activating pro-apoptotic pathways (e.g., mitochondrial apoptosis), while S1P promotes tumor proliferation, angiogenesis, and chemotherapy resistance by activating the PI3K/AKT pathway and inhibiting apoptosis and autophagy (26–28). Notably, the bidirectional regulation of the PI3K/AKT pathway by both Cer (inhibition) and S1P (activation) may be an important mechanism underlying tumor resistance. Further studies suggest that sphingolipid metabolic enzymes (such as ceramide synthase and sphingosine kinase) and their signaling networks play a broad role in regulating cell movement, the immune microenvironment, and metabolic reprogramming, contributing to the multi-stage process of tumorigenesis (12). Based on this, targeting sphingolipid metabolism (such as inhibiting S1P production or enhancing Cer accumulation) has emerged as an innovative strategy for cancer treatment, particularly in reversing HCC resistance and inhibiting metastasis (28). S1P, the product of SphK1 and SphK2, plays different roles: SphK1 drives HCC proliferation and angiogenesis through S1P signaling, while SphK2 has a dual role, with pro-apoptotic or pro-survival effects depending on subcellular localization and microenvironment signals. Targeting SphK1 or modulating SphK2’s subcellular distribution may offer new therapeutic strategies for HCC.

### 3.1 SphK1: a core driver of tumor growth

Research suggest that sphingosine kinase 1 (SphK1) plays a crucial role in metastasis and prognosis in cancer patients. SphK1 is not only involved in DNA damage response but is also closely associated with chemotherapy and radiation resistance. SphK1 catalyzes the production of sphingosine-1-phosphate (S1P) and forms the SphK1/S1P signaling axis, which plays a key role in cancer progression. Specifically, the SphK1/S1P axis promotes hepatocellular carcinoma (HCC) cell proliferation, survival, and migration through the activation of the PI3K/AKT/mTOR and MAPK/ERK signaling pathways (12). In tumor microenvironment regulation, SphK1 significantly enhances tumor angiogenesis by upregulating vascular endothelial growth factor (VEGF) expression, providing metabolic support for HCC. Experiments have confirmed that inhibition of VEGF-A-mediated angiogenesis can effectively suppress tumor growth in xenograft models (29). The absence of S1P directly impairs vascular development, highlighting the central role of this axis in blood vessel-dependent tumor growth.

Further research reveals that glycochenodeoxycholate (GCDA) may enhance HCC cell proliferation, metabolic activity, and resistance by upregulating S1PR2 receptor expression and activating the PI3K/AKT/mTOR pathway (30). Notably, activation of the SphK1/S1P/S1PR2 axis is closely associated with the malignant phenotype of HCC. Its dual effects include: on one hand, inhibiting SphK1 downregulates NF-κB p65 activity and modulates ceramide levels [e.g., with DMS intervention (31)], inducing tumor cell apoptosis; on the other hand, SphK1 activates Rho GTPases and matrix metalloproteinases (MMPs), promoting epithelial-mesenchymal transition (EMT), thereby enhancing HCC cell invasion and distant metastasis (32). These mechanisms cooperate to form a pro-cancer network, driving HCC progression. Based on these mechanisms, targeting the SphK1/S1P/S1PR2 axis could not only suppress tumor proliferation and angiogenesis but also reverse EMT phenotypes and resistance, providing promising intervention strategies for precise HCC treatment.

### 3.2 SphK2: tumor growth in the microenvironment

SphK2 is the main isoform of SphK in the liver, accounting for 90% of its total activity (33) Studies have shown that SphK2 drives the progression of non-alcoholic fatty liver disease (NAFLD)-related hepatocellular carcinoma (HCC) through dual mechanisms. First, SphK2 affects HCC development by regulating lipid metabolic balance: SphK2 deficiency downregulates the expression of ceramide transport protein (CERT), significantly decreasing the ratio of pro-cancer sphingomyelin (SM) to anti-cancer ceramide, thereby inhibiting HCC development in NAFLD liver; conversely, overexpression of CERT can reverse this effect and restore hepatocyte proliferation, colony formation, and cell cycle progression (34). SphK2 promotes carcinogenic transformation through epigenetic mechanisms: localized in the cell nucleus, SphK2 catalyzes the production of sphingosine-1-phosphate (S1P), which enhances histone acetylation (such as modulating histone acetyltransferase activity), altering the expression of lipid metabolism-related genes and oncogenes (e.g., c-Myc), thereby driving liver lipid metabolism disorder and HCC deterioration (35). Experimental models further indicate that dysregulation of SphK2 directly induces NAFLD and participates in the carcinogenic process associated with non-alcoholic steatohepatitis (NASH) through S1P-mediated epigenetic reprogramming (36). In summary, SphK2, as an indispensable factor in the cross-regulation of lipid metabolism and epigenetics in NAFLD-HCC, offers new therapeutic strategies through targeted intervention (such as gene ablation or inhibitor application).

## 4 Clinical research progress of SphK1 and SphK2 in HCC

Sphingosine kinase 1 (SphK1) and sphingosine kinase 2 (SphK2) are key enzymes that catalyze the formation of sphingosine-1-phosphate (S1P), both playing crucial but distinct roles in the pathogenesis of hepatocellular carcinoma (HCC). Current clinical studies indicate that SphK1 is significantly upregulated in HCC tissues, and this upregulation has been confirmed by antibody-based detection techniques (37). Further studies have shown that the SphK1/S1P axis is closely related to the inflammatory microenvironment and may accelerate the progression of HCC through inflammation-mediated oncogenic mechanisms (38). Unlike SphK1, SphK2 remains understudied; nevertheless, it already appears to shape tumor fate by reprogramming transcriptional networks and cell-cycle checkpoints. Notably, in vitro experiments and xenograft models have demonstrated the potential of SphK2 to inhibit HCC, recent evidence reveals that it mediates resistance to regorafenib (a standard second-line treatment drug for advanced HCC) by activating the NF-κB/STAT3 signaling pathway, suggesting that the SphK2/S1P axis may exert oncogenic effects in the HCC microenvironment (39). Notably, although *in vitro* experiments and xenograft models have demonstrated the potential of SphK2 to inhibit HCC, recent evidence reveals that it mediates resistance to regorafenib (a standard second-line treatment drug for advanced HCC) by activating the NF-κB/STAT3 signaling pathway, suggesting that the SphK2/S1P axis may exert oncogenic effects in the HCC microenvironment.

SphK1 and SphK2 are key enzymes that catalyze the production of sphingosine-1-phosphate (S1P), playing important roles in the occurrence and progression of hepatocellular carcinoma (HCC). SphK1 is highly expressed in HCC tissues, while SphK2, although less studied in HCC, can influence tumor cell behavior through regulation of gene expression and cell cycle progression.

### 4.1 Research on SphK1 inhibitors

The central role of SphK1 in HCC metastasis has been elucidated through multidimensional clinical and experimental studies. Clinical evidence shows that SphK1 mRNA expression is significantly upregulated in tumor tissues of HCC patients, and its expression level is positively correlated with tumor progression and poor prognosis. Mechanistic studies have revealed that SphK1 catalyzes the phosphorylation of sphingosine to form sphingosine-1-phosphate (S1P), activating endothelial differentiation gene (EDG1/S1PR1) receptors and directly promoting the migration and invasion of hepatocellular carcinoma cells. Inhibition of SphK1 activity (by siRNA or specific inhibitors) significantly reduces the metastatic phenotype of tumor cells, confirming that the SphK1/S1P/EDG1 signaling axis is a key regulatory hub for HCC metastasis (40). It is worth noting that although both SphK1 and S1P lyase (SGPL1) mRNA are upregulated in HCC, the net level of S1P is decreased, which may be related to the imbalance of sphingolipid metabolism in the tumor microenvironment. This dynamic change suggests that targeting the SphK1/S1P axis requires comprehensive consideration of the complexity of the metabolic network (41).

The development of SphK1 inhibitors provides a new direction for HCC treatment. Early broad-spectrum inhibitors such as N, N-dimethyl-D-erythro-sphingosine (DMS) inhibit SphK1/2 activity competitively, blocking S1P production and inducing cancer cell apoptosis, but their lack of subtype specificity limits clinical application (42, 43). In recent years, the discovery of highly selective SphK1 inhibitors (such as PF-543) has significantly improved the precision of targeted therapy: PF-543 efficiently inhibits S1P production in whole blood and exhibits significant antitumor activity in preclinical models of glioblastoma and acute myeloid leukemia (44, 45). In addition, a novel inhibitor SKI-178, by dual inhibition of SphK1 and S1P lyase, exhibits stronger proliferation-inhibitory effects in various cancer cell lines, providing a potential strategy for overcoming HCC drug resistance (46). These studies not only systematically reveal the pro-metastatic mechanisms of SphK1 but also lay a theoretical and practical foundation for the development of novel targeted drugs based on the SphK1/S1P axis.

### 4.2 Research on SphK2 inhibitors

Recent studies have revealed the unique role of SphK2 in liver cancer therapy. Functional studies have shown that knockdown of SphK2 expression or use of selective inhibitors can significantly inhibit cancer cell proliferation, migration, and invasion, with mechanisms involving induction of apoptosis and enhanced chemotherapy sensitivity (47). Notably, targeted inhibition of SphK2 exhibits more significant anticancer effects than SphK1 in various cancer cell lines, and gene knockout experiments have confirmed that the anticancer effect of SphK2 deletion is far superior to that of SphK1 or dual enzyme inhibition (48). Mechanistically, SphK2-generated S1P binds to human telomerase reverse transcriptase (hTERT) at the periphery of the nucleus, stabilizing telomerase activity via allosteric phosphorylation mimicry, thereby maintaining telomere length and promoting tumor growth, suggesting that targeting the SphK2/S1P-hTERT axis may be a novel anticancer strategy (49).

In the treatment of hepatocellular carcinoma (HCC), SphK2 inhibitors show important potential. Preclinical studies have demonstrated that the selective SphK2 inhibitor ABC294640 not only inhibits tumor growth as a monotherapy by downregulating the MAPK/ERK signaling pathway but also synergistically reduces phosphorylated ERK (p-ERK) levels when combined with the multikinase inhibitor sorafenib, enhancing pro-apoptotic effects and delaying tumor progression (50). In the treatment of hepatocellular carcinoma (HCC), SphK2 inhibitors show important potential. Preclinical studies have demonstrated that the selective SphK2 inhibitor ABC294640 not only inhibits tumor growth as a monotherapy by downregulating the MAPK/ERK signaling pathway but also synergistically reduces phosphorylated ERK (p-ERK) levels when combined with the multikinase inhibitor sorafenib, enhancing pro-apoptotic effects and delaying tumor progression (39). These findings highlight that SphK2 is not only an independent target for HCC treatment but also an important combinatorial target to optimize existing therapies (such as sorafenib and regorafenib), with its clinical translational value urgently requiring validation through subsequent studies.

## 5 Potential of SphK1 and SphK2 as therapeutic targets for HCC

As important therapeutic targets for HCC, dual inhibitors of SphK1/2 have shown significant potential in both basic research and combination therapy. Studies have shown that the SphK1/2 inhibitor SKI-II, when combined with 5-fluorouracil (5-FU), can synergistically inhibit proliferation, migration, and colony formation of HepG2 cells at low sub-toxic concentrations and significantly induce apoptosis. Mechanistic research indicates that this synergistic effect is achieved by blocking FAK-regulated IGF-1R activity and downregulating osteopontin expression, thereby inhibiting the sirtuin1/p38 MAPK-mediated NF-κB signaling pathway (51). Notably, the novel inhibitor SKI-349 exerts antitumor effects by downregulating key nodes of the AKT/mTOR signaling pathway (e.g., the ratios of p-AKT/AKT and p-mTOR/mTOR), and it produces synergistic cytotoxicity when combined with sorafenib (52). These findings provide experimental evidence for combination therapy strategies based on targeting sphingolipid metabolism.

At the level of pathological mechanisms, the SphK/S1P signaling axis plays a multidimensional regulatory role in the occurrence and development of HCC. This axis not only participates in the regulation of cellular activities during hepatitis virus infection but also constitutes a major contributing factor to hepatocarcinogenesis (53). In addition, the SphK/S1P signaling axis can influence hepatic lipid metabolism via the mTORC2 complex. In the SphK1/S1P signaling axis, mTOR acts as a downstream pathway driving abnormal proliferation, survival, and migration of HCC cells. As one of the major complexes of the mTOR pathway, mTORC2 has been implicated in studies where SphK2 was shown to promote VLDL secretion by maintaining SNARE complex stability. Inhibition of SphK2 can disrupt lipid efflux in hepatocellular carcinoma cells and induce lipotoxic accumulation, suggesting that the SphK2-mTORC2 axis may serve as a novel target for metabolic reprogramming in liver cancer (54). Furthermore, TNF-*α* signal transduction mediated by SphK can be activated by antitumor immune surveillance and immunotherapy to induce the TRAIL pathway. Inhibition of the SphK/S1P axis may help reactivate the pro-apoptotic effects of the TNF superfamily (55). Based on existing studies, combining SphK inhibitors with anti-TNF therapies, through dual targeting of intrinsic apoptotic resistance and the immunosuppressive microenvironment in HCC cells, offers a new strategy to overcome treatment resistance. Future research directions may include: optimization of treatment sequencing, such as using SphK inhibitors first to relieve immune suppression followed by anti-TNF therapy; combination with immune checkpoint inhibitors to form a multidimensional approach of “apoptosis induction and immune activation”; and development of precision therapies for HCC subtypes with concomitant lipid metabolism abnormalities (accounting for 30–40%). This “metabolism-immunity” crosstalk-based regulatory strategy may not only prolong patient survival but also provide a new paradigm for liver cancer treatment, though its clinical translation still requires further validation.

## 6 Conclusion

Progression of HCC, SphK1 and SphK2 play key roles by catalyzing the production of S1P. SphK1 promotes tumor cell proliferation, migration, and angiogenesis by activating signaling pathways such as PI3K/AKT/mTOR and MAPK/ERK. Its mediation of the S1P/S1PR2 axis further facilitates epithelial-mesenchymal transition (EMT) and chemotherapy resistance, and it can also exacerbate immune suppression in the tumor microenvironment by regulating the NF-κB inflammatory pathway. SphK2, on the other hand, plays a central role in lipid metabolism homeostasis and epigenetic regulation and is associated with HCC progression and resistance to regorafenib.

In terms of therapeutic target research, SphK1 inhibitors (such as PF-543 and SKI-178) significantly suppress tumor progression by selectively inhibiting activity or jointly targeting S1P lyase. SphK2 inhibitors (such as ABC294640) exhibit potent monotherapy and synergistic antitumor effects with sorafenib in preclinical models, with especially notable potential in reversing drug resistance.

Future research elucidate the subtype specificity of SphK1/SphK2 in different HCC types (e.g., virus-related vs. metabolism-related), continue to explore the core mechanisms of SphK2, validate its efficacy in primary HCC models, develop highly selective inhibitors to reduce toxicity, investigate optimized sequential treatment strategies, and promote clinical trials of combination therapies based on metabolism-immunity crosstalk (e.g., SphK inhibitors combined with PD-1/PD-L1 inhibitors).

Additionally, dynamic changes in serum S1P and ceramides may serve as biomarkers to aid early diagnosis and therapeutic efficacy prediction. Although significant progress has been made in mechanistic studies and targeted therapy of SphK1/SphK2, integrating multi-omics data with clinical practice is still required to advance the implementation of precision therapeutic strategies.

## References

[1] Llovet JM Kelley RK Villanueva A Singal AG Pikarsky E Roayaie S. Hepatocellular carcinoma. Nat Rev Dis Primers. (2021) 7:6. doi: 10.1038/s41572-020-00240-3, PMID: 33479224 PMC13537433

[2] Sung H Ferlay J Siegel RL Laversanne M Soerjomataram I Jemal A. Global cancer statistics 2020: GLOBOCAN estimates of incidence and mortality worldwide for 36 cancers in 185 countries. CA Cancer J Clin. (2021) 71:209–49. doi: 10.3322/caac.21660, PMID: 33538338

[3] McGlynn KA Petrick JL London WT. Global epidemiology of hepatocellular carcinoma: an emphasis on demographic and regional variability. Clin Liver Dis. (2015) 19:223–38. doi: 10.1016/j.cld.2015.01.001, PMID: 25921660 PMC4712629

[4] Kulik L El-Serag HB. Epidemiology and management of hepatocellular carcinoma. Gastroenterology. (2019) 156:477–491.e1. doi: 10.1053/j.gastro.2018.08.065, PMID: 30367835 PMC6340716

[5] Garcia-Martinez I Santoro N Chen Y Hoque R Ouyang X Caprio S. Hepatocyte mitochondrial DNA drives nonalcoholic steatohepatitis by activation of TLR9. J Clin Invest. (2016) 126:859–64. doi: 10.1172/JCI83885, PMID: 26808498 PMC4767345

[6] De Gaetano A Solodka K Zanini G Selleri V Mattioli AV Nasi M. Molecular mechanisms of mtDNA-mediated inflammation. Cells. (2021) 10:2898. 34831121 10.3390/cells10112898 PMC8616383

[7] Zhang X Wu X Hu Q Wu J Wang G Hong Z. Mitochondrial DNA in liver inflammation and oxidative stress. Life Sci. (2019) 236:116464. doi: 10.1016/j.lfs.2019.05.020, PMID: 31078546

[8] Hii LW Chung FFL Mai CW Ng PY Leong CO. Sphingosine kinase 1 signaling in breast cancer: a potential target to tackle breast cancer stem cells. Front Mol Biosci. (2021) 8:748470. doi: 10.3389/fmolb.2021.748470, PMID: 34820423 PMC8606534

[9] Rostami N Nikkhoo A Ajjoolabady A Azizi G Hojjat-Farsangi M Ghalamfarsa G. S1PR1 as a novel promising therapeutic target in cancer therapy. Mol Diagn Ther. (2019) 23:467–87. doi: 10.1007/s40291-019-00401-5, PMID: 31115798

[10] Pyne S Adams DR Pyne NJ. Sphingosine 1-phosphate and sphingosine kinases in health and disease: recent advances. Prog Lipid Res. (2016) 62:93–106. doi: 10.1016/j.plipres.2016.03.001, PMID: 26970273

[11] da Silva G Milan TM Leopoldino AM. The accumulation of sphingosine kinase 2 disrupts the DNA damage response and promotes resistance to genotoxic agents. Gene. (2024) 897:148063. doi: 10.1016/j.gene.2023.148063, PMID: 38048970

[12] Nagahashi M Matsuda Y Moro K Tsuchida J Soma D Hirose Y. DNA damage response and sphingolipid signaling in liver diseases. Surg Today. (2016) 46:995–1005. doi: 10.1007/s00595-015-1270-8, PMID: 26514817 PMC5053096

[13] Nagahashi M Hait NC Maceyka M Avni D Takabe K Milstien S. Sphingosine-1-phosphate in chronic intestinal inflammation and cancer. Advances Biol Regul. (2014) 54:112–20. doi: 10.1016/j.jbior.2013.10.001, PMID: 24210073 PMC3946530

[14] Lacaná E Maceyka M Milstien S Spiegel S. Cloning and characterization of a protein kinase a anchoring protein (AKAP)-related protein that interacts with and regulates sphingosine kinase 1 activity. J Biol Chem. (2002) 277:32947–53. doi: 10.1074/jbc.M202841200, PMID: 12080051

[15] Alvarez SE Harikumar KB Hait NC Allegood J Strub GM Kim EY. Sphingosine-1-phosphate is a missing cofactor for the E3 ubiquitin ligase TRAF2. Nature. (2010) 465:1084–8. doi: 10.1038/nature09128, PMID: 20577214 PMC2946785

[16] Pitson SM. Regulation of sphingosine kinase and sphingolipid signaling. Trends Biochem Sci. (2011) 36:97–107. doi: 10.1016/j.tibs.2010.08.001, PMID: 20870412

[17] Takabe K Kim RH Allegood JC Mitra P Ramachandran S Nagahashi M. Estradiol induces export of sphingosine 1-phosphate from breast cancer cells via ABCC1 and ABCG2. J Biol Chem. (2010) 285:10477–86. doi: 10.1074/jbc.M109.064162, PMID: 20110355 PMC2856255

[18] Strub GM Paillard M Liang J Gomez L Allegood JC Hait NC. Sphingosine-1-phosphate produced by sphingosine kinase 2 in mitochondria interacts with prohibitin 2 to regulate complex IV assembly and respiration. FASEB J. (2011) 25:600–12. doi: 10.1096/fj.10-167502, PMID: 20959514 PMC3023391

[19] Ding G Sonoda H Yu H Kajimoto T Goparaju SK Jahangeer S. Protein kinase D-mediated phosphorylation and nuclear export of sphingosine kinase 2. J Biol Chem. (2007) 282:27493–502. doi: 10.1074/jbc.M701641200, PMID: 17635916

[20] Cheng JC Wang EY Yi Y Thakur A Tsai SH Hoodless PA. S1P stimulates proliferation by upregulating CTGF expression through S1PR2-mediated YAP activation. Molec Cancer Res. (2018) 16:1543–55. doi: 10.1158/1541-7786.MCR-17-0681, PMID: 29903770

[21] Spiegel S Milstien S. Sphingosine-1-phosphate: an enigmatic signalling lipid. Nat Rev Mol Cell Biol. (2003) 4:397–407. doi: 10.1038/nrm1103, PMID: 12728273

[22] Chen H Wang J Zhang C Ding P Tian S Chen J. Sphingosine 1-phosphate receptor, a new therapeutic direction in different diseases. Biomed Pharmacother. (2022) 153:113341. doi: 10.1016/j.biopha.2022.113341, PMID: 35785704

[23] Blaho VA Hla T. An update on the biology of sphingosine 1-phosphate receptors. J Lipid Res. (2014) 55:1596–608. doi: 10.1194/jlr.R046300, PMID: 24459205 PMC4109755

[24] Camerer E Regard JB Cornelissen I Srinivasan Y Duong DN Palmer D. Sphingosine-1-phosphate in the plasma compartment regulates basal and inflammation-induced vascular leak in mice. J Clin Invest. (2009) 119:1871–9. 19603543 10.1172/JCI38575 PMC2701879

[25] Henry B Möller C Dimanche-Boitrel MT Gulbins E Becker KA. Targeting the ceramide system in cancer. Cancer Lett. (2013) 332:286–94. doi: 10.1016/j.canlet.2011.07.010, PMID: 21862212

[26] Grammatikos G Schoell N Ferreirós N Bon D Herrmann E Farnik H. Serum sphingolipidomic analyses reveal an upregulation of C16- ceramide and sphingosine-1-phosphate in hepatocellular carcinoma. Oncotarget. (2016) 7:18095–105. doi: 10.18632/oncotarget.7741, PMID: 26933996 PMC4951274

[27] Morales A Lee H Goñi FM Kolesnick R Fernandez-Checa JC. Sphingolipids and cell death. Apoptosis. (2007) 12:923–39. doi: 10.1007/s10495-007-0721-0, PMID: 17294080

[28] Oskouian B Saba JD. Cancer treatment strategies targeting sphingolipid metabolism. Adv Exp Med Biol. (2010) 688:185–205. doi: 10.1007/978-1-4419-6741-1_13, PMID: 20919655 PMC3076281

[29] Visentin B Vekich JA Sibbald BJ Cavalli AL Moreno KM Matteo RG. Validation of an anti-sphingosine-1-phosphate antibody as a potential therapeutic in reducing growth, invasion, and angiogenesis in multiple tumor lineages. Cancer Cell. (2006) 9:225–38. doi: 10.1016/j.ccr.2006.02.023, PMID: 16530706

[30] Wang G Zhang X Zhou Z Song C Jin W Zhang H. Sphingosine 1-phosphate receptor 2 promotes the onset and progression of non-alcoholic fatty liver disease-related hepatocellular carcinoma through the PI3K/AKT/mTOR pathway. Discov Oncol. (2023) 14:4. doi: 10.1007/s12672-023-00611-8, PMID: 36631680 PMC9834486

[31] Chen K Pan Q Gao Y. DMS triggers apoptosis associated with the inhibition of SPHK1/NF-κB activation and increase in intracellular Ca2+ concentration in human cancer cells. Int J Mol Med. (2014) 33:17–24. doi: 10.3892/ijmm.2013.1541, PMID: 24173614 PMC3868491

[32] Yokota T Nojima H Kuboki S Yoshitomi H Furukawa K Takayashiki T. Sphingosine-1-phosphate receptor-1 promotes vascular invasion and EMT in hepatocellular carcinoma. J Surg Res. (2021) 259:200–10. doi: 10.1016/j.jss.2020.11.044, PMID: 33307511

[33] Allende ML Sasaki T Kawai H Olivera A Mi Y van Echten-Deckert G. Mice deficient in sphingosine kinase 1 are rendered lymphopenic by FTY720. J Biol Chem. (2004) 279:52487–92. doi: 10.1074/jbc.M406512200, PMID: 15459201

[34] Liu XT Chung LH Liu D Chen J Huang Y Teo JD. Ablation of sphingosine kinase 2 suppresses fatty liver-associated hepatocellular carcinoma via downregulation of ceramide transfer protein. Oncogene. (2022) 11:67. doi: 10.1038/s41389-022-00444-0, PMID: 36333295 PMC9636415

[35] Zanini G Selleri V Mattioli AV Nasi M Pinti M. Regulation of histone acetylation in the nucleus by sphingosine-1-phosphate. Science. (2009) 325:1254–7. 19729656 10.1126/science.1176709 PMC2850596

[36] Nagahashi M Takabe K Liu R Peng K Wang X Wang Y. Conjugated bile acid-activated S1P receptor 2 is a key regulator of sphingosine kinase 2 and hepatic gene expression. Hepatology (baltimore, Md). (2015) 61:1216–26. 10.1002/hep.27592 PMC4376566 25363242

[37] Reynolds GM Visentin B Sabbadini R. Immunohistochemical detection of sphingosine-1-phosphate and sphingosine kinase-1 in human tissue samples and cell lines. Methods Mol Biol. (2018) 1697:43–56. 28560513 10.1007/7651_2017_44

[38] Pyne NJ McNaughton M Boomkamp S MacRitchie N Evangelisti C Martelli AM. Role of sphingosine 1-phosphate receptors, sphingosine kinases and sphingosine in cancer and inflammation. Advances Biol Regul. (2016) 60:151–9. doi: 10.1016/j.jbior.2015.09.001, PMID: 26429117

[39] Shi W Zhang S Ma D Yan D Zhang G Cao Y. Targeting SphK2 reverses acquired resistance of regorafenib in hepatocellular carcinoma. Front Oncol. (2020) 10:694. doi: 10.3389/fonc.2020.00694, PMID: 32670862 PMC7327090

[40] Bao M Chen Z Xu Y Zhao Y Zha R Huang S. Sphingosine kinase 1 promotes tumour cell migration and invasion via the S1P/EDG1 axis in hepatocellular carcinoma. Liver Int. (2012) 32:331–8. doi: 10.1111/j.1478-3231.2011.02666.x, PMID: 22098666

[41] Sato M Ikeda H Uranbileg B Kurano M Saigusa D Aoki J. Sphingosine kinase-1, S1P transporter spinster homolog 2 and S1P2 mRNA expressions are increased in liver with advanced fibrosis in human. Sci Rep. (2016) 6:32119. 27562371 10.1038/srep32119 PMC4999825

[42] Yatomi Y Ruan F Megidish T Toyokuni T Hakomori S Igarashi Y. N,N-dimethylsphingosine inhibition of sphingosine kinase and sphingosine 1-phosphate activity in human platelets. Biochemistry. (1996) 35:626–33. doi: 10.1021/bi9515533, PMID: 8555236

[43] Endo K Igarashi Y Nisar M Zhou QH Hakomori S. Cell membrane signaling as target in cancer therapy: inhibitory effect of N,N-dimethyl and N,N,N-trimethyl sphingosine derivatives on in vitro and in vivo growth of human tumor cells in nude mice. Cancer Res. (1991) 51:1613–8. 1998952

[44] Schnute ME McReynolds MD Kasten T Yates M Jerome G Rains JW. Modulation of cellular S1P levels with a novel, potent and specific inhibitor of sphingosine kinase-1. Biochem J. (2012) 444:79–88. doi: 10.1042/BJ20111929, PMID: 22397330

[45] Kapitonov D Allegood JC Mitchell C Hait NC Almenara JA Adams JK. Targeting sphingosine kinase 1 inhibits akt signaling, induces apoptosis, and suppresses growth of human glioblastoma cells and xenografts. Cancer Res. (2009) 69:6915–23. doi: 10.1158/0008-5472.CAN-09-0664, PMID: 19723667 PMC2752891

[46] Hengst JA Wang X Sk UH Sharma AK Amin S Yun JK. Development of a sphingosine kinase 1 specific small-molecule inhibitor. Bioorg Med Chem Lett. (2010) 20:7498–502. doi: 10.1016/j.bmcl.2010.10.005, PMID: 21050755

[47] Gao P Smith CD. Ablation of sphingosine kinase-2 inhibits tumor cell proliferation and migration. Molec Cancer Res. (2011) 9:1509–19. doi: 10.1158/1541-7786.MCR-11-0336, PMID: 21896638 PMC3219805

[48] Gao P Peterson YK Smith RA Smith CD. Characterization of isoenzyme-selective inhibitors of human sphingosine kinases. PLoS One. (2012) 7:e44543. doi: 10.1371/journal.pone.0044543, PMID: 22970244 PMC3438171

[49] Panneer Selvam S De Palma RM Oaks JJ Oleinik N Peterson YK Stahelin RV. Binding of the sphingolipid S1P to hTERT stabilizes telomerase at the nuclear periphery by allosterically mimicking protein phosphorylation. Sci Signal. (2015) 8:ra58. doi: 10.1126/scisignal.aaa4998, PMID: 26082434 PMC4492107

[50] Beljanski V Lewis CS Smith CD. Antitumor activity of sphingosine kinase 2 inhibitor ABC294640 and sorafenib in hepatocellular carcinoma xenografts. Cancer Biol Ther. (2011) 11:524–34. doi: 10.4161/cbt.11.5.14677, PMID: 21258214 PMC3087901

[51] Grbčić P Tomljanović I Klobučar M Kraljević Pavelić S Lučin K Sedić M. Dual sphingosine kinase inhibitor SKI-II enhances sensitivity to 5-fluorouracil in hepatocellular carcinoma cells via suppression of osteopontin and FAK/IGF-1R signalling. Biochem Biophys Res Commun. (2017) 487:782–8. doi: 10.1016/j.bbrc.2017.04.100, PMID: 28433634

[52] Chen L Wang L Han Z Qin P Niu G du J. SKI-349, a sphingosine kinases 1/2 inhibitor, suppresses cell viability, invasion, and AKT/mTOR signaling pathway, and shows synergistic cytotoxic effects with sorafenib in hepatocellular carcinoma. Tohoku J Exp Med. (2024) 262:173–80. doi: 10.1620/tjem.2023.J100, PMID: 38123304

[53] Zhang L Liu J Xiao E Han Q Wang L. Sphingosine-1-phosphate related signalling pathways manipulating virus replication. Rev Med Virol. (2023) 33:e2415. doi: 10.1002/rmv.2415, PMID: 36597202

[54] Zhang S Li G He L Wang F Gao M Dai T. Sphingosine kinase 2 deficiency impairs VLDL secretion by inhibiting mTORC2 phosphorylation and activating chaperone-mediated autophagy. Cell Death Differ. (2025) 10.1038/s41418-025-01507-6 PMC12500862 40200091

[55] Sukocheva OA Neganova ME Aleksandrova Y Burcher JT Chugunova E Fan R. Signaling controversy and future therapeutical perspectives of targeting sphingolipid network in cancer immune editing and resistance to tumor necrosis factor-α immunotherapy. Cell Commun Signal. (2024) 22:251. doi: 10.1186/s12964-024-01626-6, PMID: 38698424 PMC11064425

